# Mean platelet volume (MPV): new diagnostic indices for co-morbidity of tuberculosis and diabetes mellitus

**DOI:** 10.1186/s12879-021-06152-1

**Published:** 2021-05-20

**Authors:** Feifan Xu, Shengyan Qu, Lin Wang, Yongwei Qin

**Affiliations:** 1 Department of Pathogen Biology, School of Medicine, Nantong University, 19 Qixiu Road, Nantong, 226001 Jiangsu P.R. China; 2 Department of Clinical Laboratory, The Sixth Peoples Hospital of Nantong, 500 Yonghe Road, Nantong, 226011 Jiangsu P.R. China; 3 Department of Cardiothoracic Surgery, Affiliated Hospital of Nantong University, Nantong, 226001 Jiangsu P.R. China

**Keywords:** Tuberculosis, Diabetes mellitus, Diagnosis indices, Mean platelet volume, Plateletcrit

## Abstract

**Background:**

Tuberculosis (TB) and type 2 diabetes mellitus (DM) are global health diseases with high morbidity and mortality. Few studies have focused on platelet indices in TB-DM coinfection patients. The objective of this work was to analyze the platelet indices in TB, DM and TB-DM patients to assess the predictive value of the platelet index for the risk of these diseases.

**Methods:**

In total, 246 patients admitted to our hospital were distributed into three groups (113TB, 59 DM and 74TB+DM). A total of 133 individuals were also recruited as healthy controls (HC). Platelet indices, namely, platelet count (PC), mean platelet volume (MPV), plateletcrit (PCT) and platelet distribution width (PDW), were compared among the four groups, and the relationship with inflammatory markers was explored by using statistical software.

**Results:**

Our study discovered that MPV and PCT were significantly downregulated in TB+DM patients (9.951.25fL, 0.200.05%, *P*<0.0001, *P*=0.0121, separately) compared with DM individuals (10.921.17fL, 0.220.04%). Moreover, the changes in MPV were significantly higher in TB+DM patients (9.951.25fL, *P*=0.0041) than in TB patients (9.421.01fL). No differences were found in PLT and PDW among the four groups (*P*>0.05). The sensitivity and specificity of MPV in the differential diagnosis of DM patients vs TB+DM patients were 64.9 and 66.1% (*P*<0.0001), respectively, and the sensitivity and specificity of MPV between TB patients and TB+DM patients was 60.8 and 66.4%, respectively (*P*=0.003). MPV improved the diagnosis sensitivity when it was combined with clinical parameters, such as fasting blood glucose in DM and *Mycobacterium tuberculosis* culture result in TB (76.3% vs 64.9, 72.6% vs 60.8%, *P*<0.0001, *P*=0.001, respectively). In addition, the sensitivity and specificity of PCT in the differential diagnosis of DM patients vs TB+DM patients were 69.5 and 59.4%, respectively (*P*=0.008). PCT improved the diagnosis sensitivity when combined with fasting blood glucose in DM (72.9% vs 64.9%, *P*=0.004). In addition, MPV was linked to CRP (C-reactive protein) and ESR (erythrocyte sedimentation rate) in the TB+DM patients (*r*=0.3203, *P*=0.0054, *r*=0.2504, *P*=0.0307) but PCT was not (*r*=0.1905, *r*=0.008675, *P*>0.05, respectively).

**Conclusions:**

Our research shows that MPV and PCT might be good clinical laboratory markers to distinguish TB+DM patients from TB or DM individuals, thus providing support for earlier clinical diagnosis, prevention, and therapy.

## Background

Tuberculosis (TB) is a global health disease caused by infection with *Mycobacterium tuberculosis* (MTB), despite its advanced developments in diagnosis and therapy [1]. In 2018, 10.0 million people were diagnosed with tuberculosis worldwide. China has the second largest tuberculosis epidemic worldwide, behind India, with more than 1.3 million new cases of tuberculosis every year. Ending the TB epidemic by 2030 is among the health targets of the Sustainable Development Goals.

Studies have demonstrated that some diseases accelerate TB occurrence and development [2]. Type 2 diabetes mellitus (DM) has been verified as one of the threatening risk factors for TB, and patients have three times the risk of developing TB compared to nondiabetic patients due to pathogenic mechanisms and metabolic factors [3,5]. The DM prevalence among patients with TB in diversified low-income and middle-income countries ranged from 1.8 to 45%, and TB prevalence among individuals with DM varied from 0.1 to 6.0% [6]. A national survey performed in 2010 found that the prevalence of DM was 11.2% (95% confidence interval 10.5 to 11.9%) in China [7]. Nearly 40% of TB cases in China and India are diabetes-related [8]. Clinically, DM facilitates TB development and hampers TB therapy, while conversely, TB impairs blood glucose control [9]. In addition, some studies have shown that proper blood glucose control will have a positive effect on reducing TB morbidity and mortality [10,12]. Published studies on the full prevalence of tuberculosis in Chinese patients with diabetes are far from sufficient. On the other hand, global evidence on the relationship between tuberculosis risk and blood glucose is still inconsistent [13,15]. To a great extent, TB and DM diagnoses are based on definitive detections, including clinical symptoms and characteristic X-ray and laboratory examinations, with separate limitations [16]. Given the increasing global burden of DM, clinical and public health interventions against this co-epidemic may lead to better tuberculosis prevention and treatment. However, due to the lack of established screening standards and methods, the effectiveness and reliability of screening for tuberculosis in patients with DM are not convincing [17]. In addition, few studies have focused on diagnostic markers to predict whether TB or DM will develop into a TB-DM coinfection (TB+DM). Thus, finding a feasible and cost-effective marker from laboratory reports for the early prevention and control of TB+DM is essential.

Platelets are enucleate cells and have critical roles in thrombosis, homeostasis and the inflammatory response [18]. When the internal environment is changed, platelet morphology may be altered and play a role in certain platelet-associated parameters, mainly including platelet count (PC), plateletcrit (PCT), platelet distribution width (PDW) and mean platelet volume (MPV). MPV detection and assays are generally valid in the clinic as they are routinely performed. MPV is increased in intestinal diseases, respiratory diseases, cardiovascular diseases, cerebral stroke, several cancers and diabetes. Conversely, MPV is decreased in ulcerative colitis, neoplasm, Systemic Lupus Erythematosus (SLE) and tuberculosis [19]. The mechanisms for an increased MPV are not well clarified. Several factors may influence MPV levels, including genetic variations, applied treatment drugs, lifestyle (diet, smoking, alcohol consumption, and physical activity), pre- and analytical procedures, hormonal profile, age, gender and race/ethnicity [19, 20]. Studies have demonstrated that changes in PC, especially during the process of TB infection, might be correlated with the mortality and severity of the infection [21, 22]. MPV is a marker reflecting the average size of platelets present in various diseases, such as DM, metabolic syndrome and TB [23]. However, the role of MPV in TB is disputed. A study conducted by Gunluoglu et al. suggested that MPV, as an inflammation marker, decreased in active pulmonary tuberculosis related to the formation of microthrombi in TB cavities [24]. Conversely, Tozkoparan et al. suggested that MPV was significantly increased in patients suffering from active TB and downregulated with anti-TB treatment [21]. In addition, MPV also served as a clue for the reflection of platelet activation in DM regardless of the diabetic retinopathy stage [25]. In addition to MPV, additional platelet indices PDW and PCT calculated by PC and MPV have been reported to play roles in atherosclerosis and thrombosis, as well in TB [26]. Higher PDW and PCT values developed frequently in patients with PTB with a strong correlation between phase reactants and acute thrombocytosis [27]. However, few studies have explored the relationship between platelet-associated parameters and TB-DM coinfection patients.

The prediction of the risk of TB-DM coinfection is absolutely vital for TB and DM patients. The purpose of the study is to assess the possible relationships of TB-DM coinfection with platelet indices PC, MPV, PCT and PDW. To this end, we also determined the relationship of these parameters with inflammatory markers (CRP and ESR index). We propose new novel biomarkers for diabetes with tuberculosis for earlier diagnosis and treatment.

## Methods

### Study setting and data sources

This was a single-centre study. All data were collected in our hospital from July 2018 to August 2019. The study complied with the Declaration of Helsinki, and the Human Ethical Committee of the Sixth Peoples Hospital of Nantong approved the study protocol. We obtained informed consent from all participants involved in our study. Participants enrolled in the study were given written informed consent. In all, 379 participants were included: 133 healthy controls (HC), 113TB patients, 59 DM patients and 74TB+DM patients. HC were selected by medical examination center with no expose of MTB, no clinical characteristic of TB and the PPD test were negative. TB diagnose based on positive results of Xpert MTB/RIF (Cephaid Inc., CA, USA), BACTEC MGIT 960 rapid liquid isolate culture (Becton Dickinson, Sparks, USA) by GenoTypeH check system (Hain Lifescience, Nehren, Germany) and MTB smear confirmation by Ziehl-Neelsen acid-fast stain (Zhuhai DL biotech co,. Ltd., Guangdong, China). DM patients excluded TB were enrolled from the Endocrine Department in our hospital and diagnosed with DM previously according to a WHO-criteria. TB-DM coinfection patients were certified TB along with hyperglycemia (fasting glucose7.0mmol/L) and HbA1c6.5%. Full blood counts were carried out using Mindray BC-6900 chemistry analyzer (Shenzhen Mindray Bio-Medical Electronics Co. Ltd., China). The data of ESR (Erythrocyte sedimentation rate) and CRP (C-reactive protein) were picked from the Clinical Laboratory Department of our hospital measured by Eriline AR Linear (Barcelona, Spain) and Beckman Coulter 5800 (Tokyo, Japan). Participants were obviated if they were positive HIV examination, pregnant, Hepatitis B positive or combined with affecting the platelet indices associated diseases (i.e., cardiovascular disease, hypertension, juvenile systemic lupus erythematous (SLE), Crohns disease, cancer and others).

### Data analysis

All data processing and analyses were applied using GraphPad Prism version 5.0 software (San Diego, CA) and SPSS 17.0 software (SPSS Inc., Chicago, IL, USA). The difference between unpaired samples was analyzed using one-way ANOVA, *t*-test or chi-squared test. For the basic statistic for the cases enrolled in this study, percentiles and MeanSD were used. The area under the curve (AUC), 95% confidence interval (95% CI) sensitivity and specificity were determined by a Receiver Operating Characteristic (ROC) curve. The association between 2 quantitative variables was measured using bivariate correlation (Pearson or Spearman). All tests were two-tailed and a threshold of *P*<0.05 was perceived as statistically significant.

## Results

### Characteristics of the study population

Patient characteristics are shown in Table1. In the present study, the age ranged from 14 to 90years. On average, patients with DM (59.614.0) and TB+DM (58.112.5) were older and had a lower BMI (20.01.9, 20.62.2) than the HC (45.417.2, 20.92.1) and TB (47.017.8, 21.12.4) groups. In the present study, the total number of males among the four groups was 204 (53.83%), and the total number of females was 175 (46.17%). The number of males in the TB group was 112 (54.90%), with 92 (45.10%) in the non-TB group, and the number of females in the TB group was 75 (42.86%), with 100 in the non-TB group (57.14%). The were 78 males in the DM group (38.24%) and 126 (61.76%) in non-TB group and 55 (31.43%) females in the DM group and 120 (68.57%) in the non-TB group. All (without TB) DM patients were on anti-DM drug treatment, while 67.6% had TB+DM. TB+DM patients not on any treatment were given a definite diagnosis of DM. In addition, the increased glucose had no effect on interferon- release, TB drug resistance or TB pulmonary cavity formation (TB vs TB+DM).

**Table 1:** Patient characteristics in the four groups (*n* = 379)

Characteristic	HC	TB	DM	TB+DM	*P* value
	*n* = 133	*n* = 113	*n* = 59	*n* = 74	
Sex (male/female)	60/73	66/47	32/27	46/28	0.0497*
Age (years)	45.417.2	47.017.8	59.614.0	58.112.5	<0.0001*
BMI (kg/m^2^)	20.92.1	21.12.4	20.01.9	20.62.2	0.0110*
HbA1c (%)	NA^a^	NA	11.54.5	11.33.8	0.3915
Fasting glucose	NA	NA	13.35.5	12.33.7	0.2366
Smoking history	NA	48/113 (42.5%)	20/59 (33.9%)	38/74 (51.4%)	0.1219
TSPOT.TB (+)	NA	96/110 (87.3%)^b^	NA	42/74 (56.6%)	0.5726
DM medication	NA	NA	59/59 (100.0%)	50/74 (67.6%)	0.0011*
Insulin			10/59 (16.9%)	5/50 (10.0%)	0.0832
Metformin			42/59 (71.2%)	43/50 (86.0%)	0.5362
Others			7/59 (11.9%)	2/50 (4.0%)	0.1591
Years since DM diagnose	NA	NA			
<1			0/59 (0.0%)	11/74 (14.9%)	0.0006*
1-5			21/59 (35.6%)	32/74 (43.2%)	0.0006*
6-15			28/59 (47.5%)	28/74 (37.8%)	0.2695
>15			10/59 (16.9%)	3/74 (4.1%)	0.0070*
With TB drug resistant	NA	8/113 (7.1%)	NA	2/74 (2.7%)	0.1953
With TB pulmonary cavity	NA	32/113 (28.3%)	NA	15/74(20.0%)	0.2169

Different characteristic and statistic results among the four groups. *HC* Healthy community controls, *TB* Tuberculosis group, *DM* Diabetes group, *TB+DM* TB-DM co-morbidity group, *BMI* Body Mass Index, *HbA1c* Hemoglobin A1c, *TSPOT.TB* tuberculosis infectious T-lymphocyte spot assay; ^a^*NA* not applicable; ^b^Data available from 96/110 patients. *Significant value (*P* < 0.05)

### MPV and PCT might be new laboratory indicators in TB combined with DM patients

**Fig. 1:**
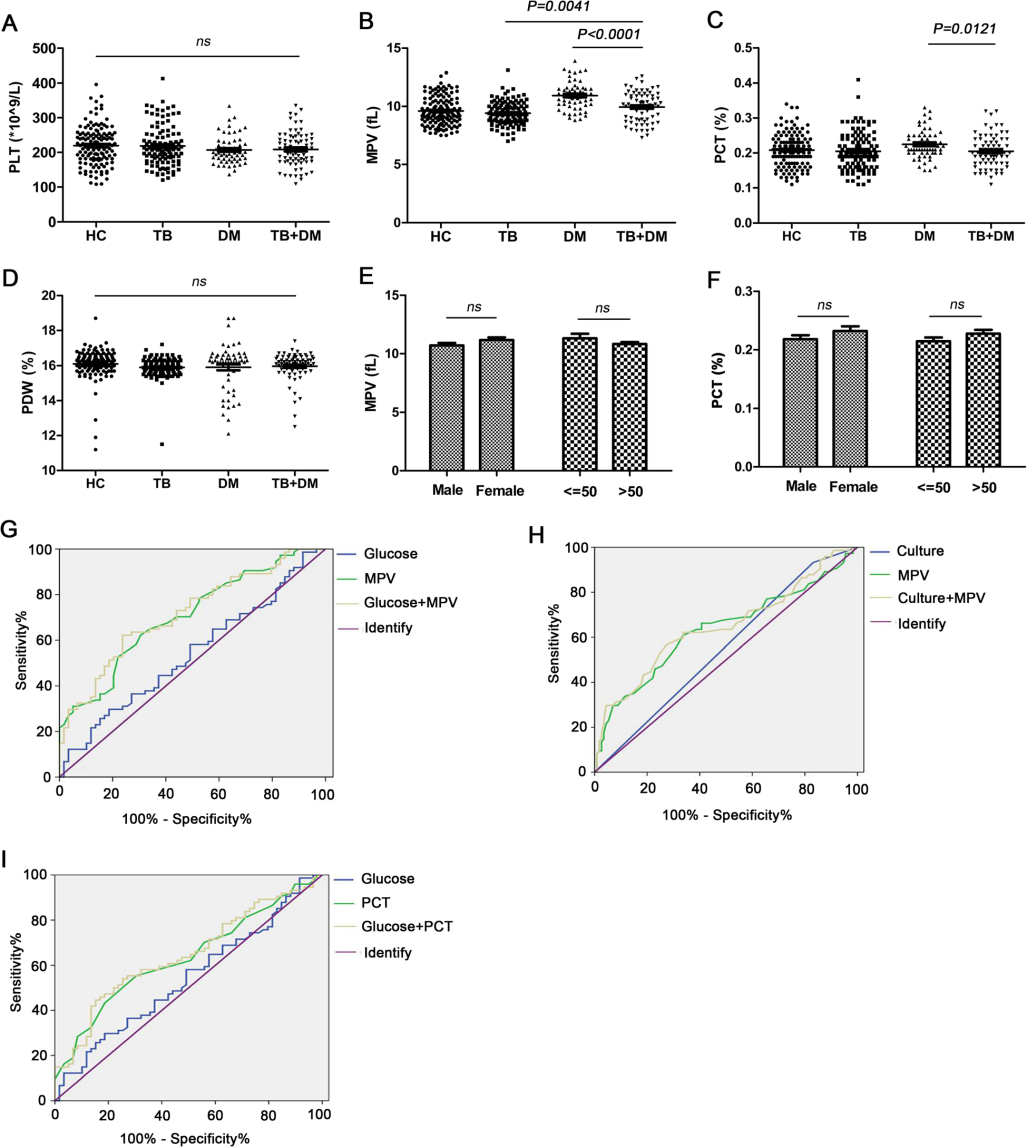
The changes of platelet related parameters in the four groups. **a**-**d** The comparison of PLT, MPV, PCT and PDW values in HC, TB, DM and TB+DM groups; **e** ROC curve for using MPV and PCT levels in the differential diagnosis of TB+DM from DM or TB. **f**-**g** MPV and PCT levels in different sex and age groups. HC, Healthy community controls; TB, Tuberculosis group; DM, Diabetes group; TB+DM, TB-DM coinfection group. PLT, Platelet; MPV, Mean platelet volume; PDW, Platelet distribution width; PCT, Plateletcrit

To investigate whether the platelet influence was involved in TB+DM patients, the changes in platelet-related parameters in the four groups were analyzed by our laboratory. As shown in the results, no differences were found in PC and PDW among the four groups (*P*>0.05, Fig.1a, d, Table2). Compared with TB (9.421.01fL), the changes in MPV were significantly increased in TB+DM patients (9.951.25fL, *P*=0.0041, Fig.1b, Table2). Compared with DM patients (10.921.17fL, 0.220.04%), the changes in MPV and PCT were significantly decreased in TB+DM patients (9.951.25fL, 0.200.05%, *P*<0.0001, *P*=0.0121, Fig.1b-c, Table2). ROC curve analysis was used for the MPV and PCT values among DM patients vs TB+DM patients and the MPV value among TB patients vs TB-DM coinfection patients. The sensitivity and specificity of MPV in the differential diagnosis of DM patients vs TB+DM patients were defined as 64.9 and 66.1%, respectively (Fig.1g, Table3, *P*<0.0001), and as 60.8 and 66.4% in the differential diagnosis of TB patients vs TB+DM patients (Fig.1h, Table3, *P*=0.003). MPV improved the diagnosis sensitivity when combined with clinical parameters such as fasting blood glucose in DM and *M. tuberculosis* culture result in TB patients (76.3% vs 64.9, 72.6% vs 60.8%, Fig.1g-h, Table3, *P*<0.0001, *P*=0.001, separately). In addition, the sensitivity and specificity of PCT in the differential diagnosis of DM patients vs TB+DM patients were 69.5 and 59.4%, respectively (Fig.1i, Table3, *P*=0.008). PCT improved the diagnosis sensitivity when combined with fasting blood glucose in DM patients (72.9% vs 64.9%, Fig.1i, Table3, *P*=0.004). ROC-related parameter data are shown in Table3. Sex and age affected MPV and PCT expression (Fig.1e-f). Thus, MPV and PCT might be laboratory markers distinguishing TB+DM patients from TB or DM patients.

**Table 2:** Comparison of platelet indices among the four groups

	HC	TB	DM	TB+DM	TB VS DM	TB VS TB+DM	DM VS TB+DM
	*n* = 133	*n* = 113	*n* = 59	*n* = 74	*P* value	*P* value	*P* value
PLT, 10^9^/L	219.455.4	218.456.9	207.040.3	208.750.1	0.3050	0.1936	0.8575
MPV, fL	9.601.21	9.421.01	10.921.17	9.951.25	<0.0001*	0.0041*	<0.0001*
PDW, %	16.10.8	15.90.6	15.91.3	16.00.8	0.6804	0.3821	0.3409
PCT, %	0.210.05	0.200.05	0.220.04	0.200.05	0.0397*	0.7066	0.0121*

Platelet associated parameters and statistic results among the four groups. *HC* Healthy community controls, *TB* Tuberculosis group, *DM* Diabetes group, *TB+DM* TB-DM co-morbidity group, *PLT* Platelet, *MPV* Mean platelet volume, *PDW* Platelet distribution width, *PCT* Plateletcrit. *Significant value (*P* < 0.05)

**Table 3:** The associated parameters of ROC in TB, DM and TB + DM groups

	Sensitivity%	Specificity%	AUC	95% CI	*P*
DM vs TB+DM group					
Glucose	52.5	50.0	0.540	0.441-0.638	0.432
MPV	64.9	66.1	0.705	0.618-0.792	<0.0001*
Glucose+MPV	76.3	62.2	0.716	0.630-0.802	<0.0001*
TB vs TB+DM group					
Culture	17.0	93.2	0.553	0.469-0.636	0.227
MPV	60.8	66.4	0.630	0.544-0.716	0.003*
Culture+MPV	72.6	56.8	0.645	0.561-0.729	0.001*
DM vs TB+DM group					
Glucose	52.5	50.0	0.540	0.441-0.638	0.432
PCT	69.5	55.4	0.635	0.541-0.728	0.008*
Glucose+PCT	72.9	55.4	0.645	0.552-0.738	0.004*

The AUC curve results of MPV and PCT in TB, DM and TB+DM groups. *TB* Tuberculosis group, *DM* Diabetes group, *TB+DM* TB-DM co-morbidity group, *Glucose* Fasting blood glucose, *Culture* mycobacterium tuberculosis culture, *PLT* Platelet, *MPV* Mean platelet volume, *PDW* Platelet distribution width, *PCT* Plateletcrit, *AUC* Area under curve, *95% CI* 95% confidence interval. *Significant value (*P* <0.05)

### MPV is associated with the course of an inflammatory condition instead of PCT

**Fig. 2:**
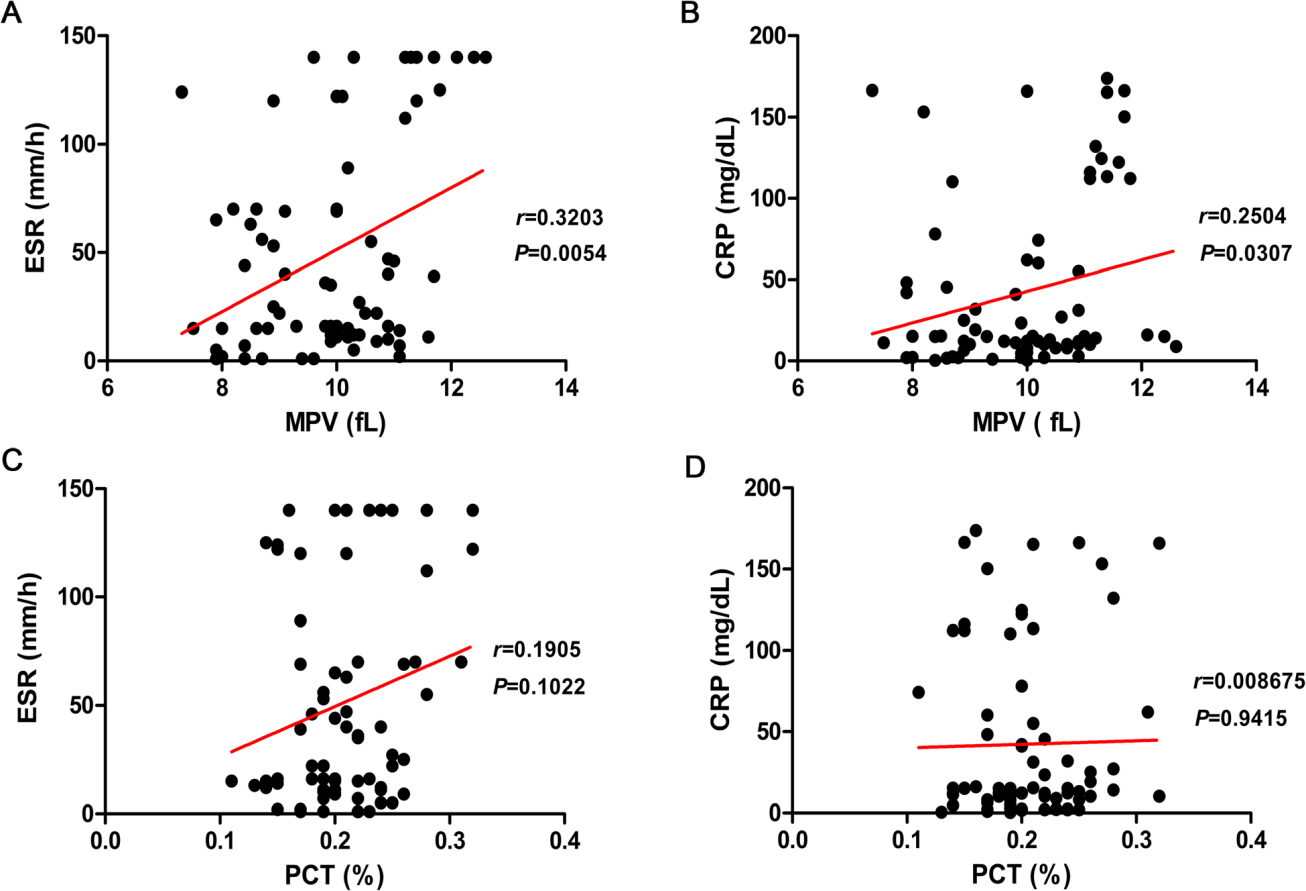
The correlation of MPV and PCT with ESR and CRP in the TB+DM group. **a** The relevance curve of MPV with ESR; **b** The relevance curve of MPV with CRP; **c** The relevance curve of PCT with ESR; **d** The relevance curve of MPV with CRP. MPV, Mean platelet volume; PCT, Plateletcrit; ESR, Erythrocyte sedimentation rate; CRP, C-reactive protein

CRP and ESR are common markers of inflammatory status. The correlation of these markers with MPV and PCT was also analyzed. MPV was associated with ESR (*r*=0.3203, *P*=0.0054, Fig.2a) and CRP (*r*=0.2504, *P*=0.0307, Fig.2b) values in the TB+DM group, while it was not associated with CRP and ESR in PCT (*r*=0.1905, *r*=0.008675, *P*>0.05, separately, Fig.2c-d). Thus, MPV might be used as a potential indicator to evaluate whether TB or DM will develop into TB-DM coinfection, and it is correlated to the inflammatory index (CRP and ESR).

## Discussion

The diagnosis of TB has always used a compound method, combining clinical symptoms, etiological examination of MTB, radiology examination and TB-associated molecular testing [28]. The diagnosis of DM is based on globally accepted criteria, relying on fasting plasma glucose measurement, 2-h postchallenge results in an oral glucose tolerance test (OGTT), or a hemoglobin A1c report [29]. Unfortunately, there are no diagnostic standards for DM-TB coinfection. The International Diabetes Foundation predicted a prevalence of DM among patients with TB at 8.8% in 2017. The global prevalence is almost twice that reported by the International Diabetes Foundation. However, Noubiap concluded that the population may recommend screening for TB patients for diabetes in high-income countries, and in low-income countries, diabetes has a lower prevalence and lower health status and fewer medical resources and screening methods and requires more research to determine the most systematic diagnostic strategies [9].

Platelet indices have been associated with multiple diseases of the immune system and hemopoietic system [30]. In our study, we found that MPV and PCT were significantly decreased in TB-DM coinfections compared with DM individuals. The indices of MPV were higher in DM combined with TB than in TB patients. The increase in MPV was correlated with ESR and CRP in DM+TB patients. For the PLT and PDW indices, there was no significant change among TB, DM, and TB combined with DM and in healthy controls. Moreover, the factors of age and gender did not significantly affect the MPV and PLT indices.

The MPV reflects platelet size and the extent of inflammation and is used to reveal the function of platelets. The platelet size is associated with the inflammatory intensity [31]. In patients infected by *M. tuberculosis*, acute phase reactants and proinflammatory cytokines affect megakaryocytes, which decrease the platelet size, and smaller platelets are delivered from the bone marrow [32], which can be used to explain the decrease in MPV. Tozkoparan E et al. found that PDW and PCT were higher in active TB patients and decreased significantly after anti-tuberculosis therapy [21]. Sahin et al. indicated that MPV in active TB patients was identical to that in healthy individuals and nonspecific pneumonia patients [23]. These results were in accordance with our findings, in that there were no significant differences between TB patients and healthy controls. Gunluoglu et al. suggested that the value of MPV was slightly decreased in TB patients, and the MPV never reflected the severity of tuberculosis [24].

ESR has also been regarded as a predictor of inflammatory and autoimmune diseases. In principle, the increase in ESR is due to changes in serum proteins, or it is due to changes in erythrocytes. The former usually includes hypergammaglobulinemia, monoclonal blood diseases, and elevated fibrinogen levels. The latter is mainly to reduce the number of erythrocytes and the size of erythrocytes [33]. Our results showed that MPV, but not PLT, was correlated with ESR in TB patients with DM. Thus, it can be deduced that, as an index of blood in patients with TB and DM, MPV may be an important hematological indicator to evaluate the risk of TB and DM along with ESR.

It is well known that DM is a metabolic dysfunction characterized by hyperglycemia, which leads to vascular complications. TB is an immemorial and common infectious disease. DM and TB coinfection is a widespread public health issue. There is still a lack of knowledge and assessment regarding whether TB makes individuals susceptible to DM. Several cross-sectional studies have displayed the relationship between TB outcome and the occurrence of hyperglycemia [34]. As DM and TB increase in low- and middle-income countries, WHO and the International Union Against Tuberculosis and Lung Disease (IUATLD) encourage the establishment of a cooperative framework that recommends bidirectional screening including TB testing among individuals with DM [35]. The WHO recommends screening for DM at the start of TB treatment. In China, the bidirectional screening program was implemented in September 2011 [36]. At the time of registration, TB patients were asked if they have diabetes and for those who deny any known disease, a random blood glucose test is performed to determine who is at risk. Patients with high blood glucose levels were randomly followed up with glucose concentration testing. These patients were willing to be screened. A total of 1213% of TB patients have diabetes, of which 3% of Chinese patients are diagnosed with previously unrecognized diabetes based on fasting blood glucose values [8].

A major aim of this study is the comparison of platelet indices (MPV and PCT) and ESR in DM, TB and DM with TB patients to assess whether patients with TB or DM are at risk for developing TB+DM. Screening for TB or DM using platelet indices may improve early TB-DM coinfection detection and diagnosis. This analytical approach meets a clinical goal and establishes a potential diagnosis standard for the clinical laboratory. There is great potential for meaningful research on DM-TB in low- and middle-income regions. Prospective studies are urgently needed to resolve the differences between DM and non-DM tuberculosis patients and TB-DM diagnosis strategies. Identifying the population at risk and then conducting bidirectional screening across the entire region should be the ultimate goal of the health authorities.

### Limitations of the study

Known limitations in this study are that it was a single-center study that neglected race and genetic variations. Moreover, there may be a lack of prospective studies with a definite diagnosis. To popularize and apply the values of MPV and ESR as diagnostic markers of TB with DM, we should further verify these indices in a multicenter clinical sample and conduct cohort studies in the near future, both in China and abroad. The mechanism of abnormal MPV levels in TB, DM and DM-TB coinfection patients has yet to be fully understood.

## Conclusions

MPV is a valuable candidate marker to screen for TB-DM coinfection risk, as the occurrence of TB developing into TB-DM coinfection will increase MPV levels, and DM developing into a DM-TB coinfection will decrease MPV levels. Moreover, MPV has a positive correlation with ESR and CRP. MPV has significant specificity and sensitivity for predicting and diagnosing DM-TB coinfection. Therefore, fasting blood testing (including glucose and MPV) should be carried out in active/suspected patients with high-risk TB or DM. It is our responsibility to take care of all TB patients, not only in the diagnosis of TB but also because they are at increased risk of diabetes. Since DM can increase the risk of TB infection and the goal of the WHO strategy to end tuberculosis is to reduce TB deaths by 90% and TB incidence by 80% by 2030, we suggest free treatment policies for DM patients in low- and middle-income countries so that DM and DM-TB coinfection can be better controlled. In conclusion, MPV has potential value as a candidate marker for dual screening algorithms. Screening for diabetic tuberculosis patients should be considered in low-income countries and should be integrated at a culturally educational level and social policy-driven behavior.

## Data Availability

The datasets used and analysed during the current study are available from the corresponding author on reasonable request.

## Abbreviations

TB: Tuberculosis
DM: Diabetes mellitus
HC: Healthy control
PC: Platelet count
MPV: Mean platelet volume
PDW: Platelet distribution width
PCT: Plateletcrit
ESR: Erythrocyte sedimentation rate
CRP: C-reactive protein
